# Pretreatment ADC is not a prognostic factor for local recurrences in head and neck squamous cell carcinoma when clinical T-stage is known

**DOI:** 10.1007/s00330-019-06426-y

**Published:** 2019-09-16

**Authors:** Boris Peltenburg, Juliette P. Driessen, Jeanine E. Vasmel, Frank A. Pameijer, Luuk M. Janssen, Chris H. J. Terhaard, Remco de Bree, Marielle E. P. Philippens

**Affiliations:** 1 Department of Radiotherapy, University Medical Center Utrecht, Heidelberglaan 100, 3584 CX Utrecht, The Netherlands; 2 Department of Head and Neck Surgical Oncology, UMC Utrecht Cancer Center, University Medical Center Utrecht, Utrecht, The Netherlands; 3 Department of Otorhinolaryngology, University Medical Center Utrecht, Utrecht, The Netherlands; 4 Department of Radiology, University Medical Center Utrecht, Utrecht, The Netherlands

**Keywords:** Diffusion magnetic resonance imaging, Head and neck neoplasms, Neoplasm recurrence, local, (Chemo)radiotherapy, Disease-free survival

## Abstract

**Objectives:**

Pretreatment identification of radio-insensitive head and neck squamous cell carcinomas (HNSCC) would affect treatment modality selection. The apparent diffusion coefficient (ADC) of a tumor could be a predictor of local recurrence. However, little is known about its prognostic value next to known factors such as clinical T-stage. The aim of the present study is to determine the added value of pretreatment ADC to clinical T-stage as a prognostic factor for local recurrence.

**Methods:**

This retrospective cohort study included 217 patients with HNSCC treated with (chemo)radiotherapy between April 2009 and December 2015. All patients underwent diffusion-weighted MRI prior to treatment. Median ADC values of all tumors were obtained using a semi-automatic delineation method. Univariate models containing ADC and T-stage were compared with a multivariable model containing both variables.

**Results:**

Fifty-eight patients experienced a local recurrence within 3 years. On average, the ADC value in the group of patients with a recurrence was 1.01 versus 1.00 (10^−3^ mm^2^/s) in the group without a recurrence. Univariate analysis showed no significant association between tumor ADC and local recurrence within 3 years after (chemo)radiotherapy (*p* = 0.09). Cox regression showed that clinical T-stage was an independent predictor of local recurrence and adding ADC to the model did not increase its performance.

**Conclusion:**

Pretreatment ADC has no added value as a prognostic factor for local recurrence to clinical T-stage.

**Key Points:**

*• Pretreatment identification of head and neck squamous cell carcinoma patients who do not benefit from (chemo)radiotherapy could improve personalized cancer care.*

*• The apparent diffusion coefficient (ADC) obtained from diffusion-weighted MRI has been reported to be a prognostic factor for local recurrence.*

*• In this study, ADC has no added value as a prognostic factor compared with clinical T-stage.*

**Electronic supplementary material:**

The online version of this article (10.1007/s00330-019-06426-y) contains supplementary material, which is available to authorized users.

## Introduction

Patients with head and neck squamous cell carcinomas (HNSCC) could benefit from reliable pretreatment identification of radio-insensitive tumors. This would enable patient selection for primary surgery, avoiding the surgical challenges and morbidity induced by previous irradiation [1].

Several studies investigated the correlation of pretreatment apparent diffusion coefficient (ADC) and local tumor recurrence after (chemo)radiotherapy ((C)RT) [2–7]. Most studies concluded that tumors with a relatively high pretreatment ADC have a higher chance of local recurrence [2–4]. However, many of these studies did not account for clinical factors such as T-stage, which is known to be an important prognostic factor for local recurrence [8]. Therefore, the aim of the current study is to find the added value of ADC to clinical T-stage as a prognostic factor for local recurrence of HNSCC.

## Methods and materials

An extended explanation of the methods used in this study can be found in the supplemental materials.

This retrospective study was approved by the institutional review board and the need for informed consent was waived. In total, 217 patients with HNSCC treated with (chemo) radiotherapy were analyzed in the study. Patients were treated between April 2009 and December 2015. All patients underwent diffusion-weighted MRI prior to treatment (Table S1). Available *b* values differed across scans with all containing at least a high (*b*800 or *b*1000 s/mm^2^) and a low (*b*0 s/mm^2^) *b* value. Median ADC values of all tumors were obtained using a semi-automatic delineation method. The outcome under investigation, local recurrence, was determined by biopsy (*n* = 40), by progression of a suspected recurrence (*n* = 11) or by death of the patient during recurrence workup (*n* = 7). All other patients were considered to have local control. Patients were followed up for 3 years. To determine the added value of ADC to clinical T-stage as a prognostic factor for local recurrence, univariable models containing ADC and T-stage were compared with a multivariable model containing both variables.

## Results

### Patients

A diagram showing the flow of patients is provided in the supplemental material (Fig. S1).

Of the 217 analyzed patients (Table 1), 14 patients (6.5%) were lost to follow-up within 3 years. Fifty-eight patients (27%) developed a local recurrence within 3 years. See S2 for an example of the DW-MRI of one of the patients.

**Table 1:** Baseline patient characteristics (*N* = 217)

Variable	*N*	%
Age in years	63 (40–87)*	
Sex		
Female	55	25
Male	162	75
Tumor site		
Larynx	69	32
Hypopharynx	35	16
Oropharynx	102	47
Oral cavity	11	5
AJCC tumor stage^a^		
T2	81	37
T3	71	33
T4a	53	24
T4b	12	6
Nodal stage		
N0	95	44
N1	26	12
N2a	2	1
N2b	53	24
N2c	41	19
HPV status oropharyngeal tumors		
Positive	18	18
Negative	62	61
Unknown	22	21
Days between MRI and start treatment*	18 (1–63)	
Treatment		
Radiotherapy only	120	55
Radiotherapy + cisplatin	66	31
Radiotherapy + cetuximab	31	14
Months of follow-up*	34 (2–102)	

^a^*AJCC*, American Joint Committee on Cancer 7th edition

*Median (range)

### Predictive value of ADC and T-stage for local control

No significant difference in tumor ADC between the groups with local recurrence and those with local control was found 3 years after (C)RT: The mean of median ADC values ± SD was 1.01 ± 0.18 (× 10^−3^ mm^2^/s) in the group with local recurrence and 1.00 ± 0.24 (× 10^−3^ mm^2^/s) (*p* = 0.72) in the group without local recurrence (S3).

The most discriminating cutoff value of ADC was 0.90 × 10^−3^ mm^2^/s. The group of patients with an ADC value higher than this value had no significant difference in the rate of local recurrence compared with the group with ADC values lower than this value (*p* = 0.09) (S4).

The rate of local recurrence was different for each T-stage; patients with T2 or T3 tumors had a significantly lower rate of tumor recurrence than the patients with T4a or T4b tumors (*p* < 0.01) (S5).

**Fig. 1:**
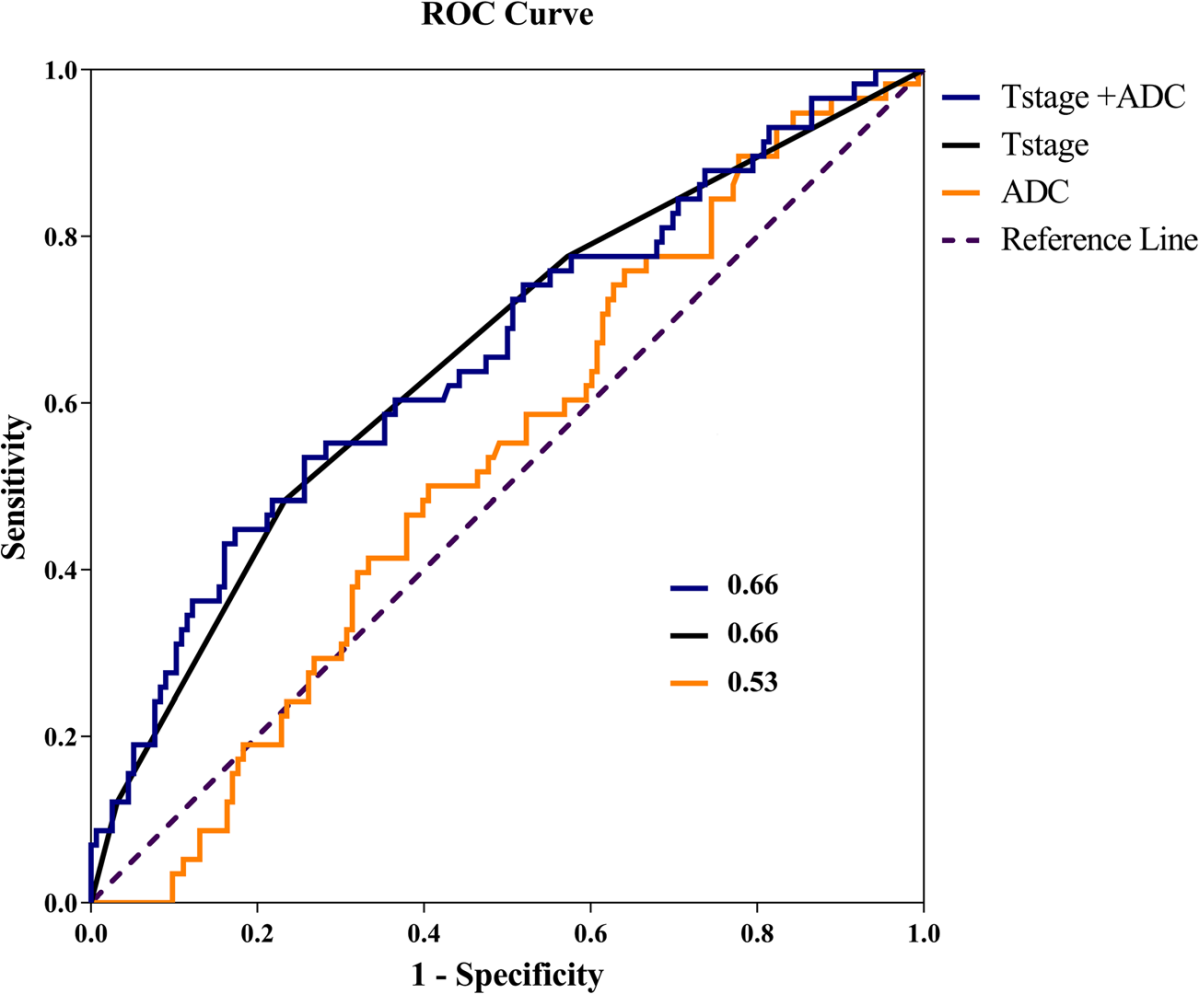
ROC curve displaying the performance of the models containing T-stage and ADC as individual parameters (black and yellow) and the performance of the model containing both variables (blue)

Cox regression showed that T-stage was associated with local recurrence (Table S2). The accuracy of the model expressed as the AUC was 0.66 (CI95% 0.57–0.74). In the multivariable Cox regression model containing both T-stage and ADC values, ADC was not independently associated with local recurrence (Table S3). The AUC of this model was 0.66 (CI95% 0.58–0.74) while the AUC based solely on ADC was 0.53 (CI95% 0.45–0.62) (Fig. 1).

### HPV and ADC

Of the 102 patients with oropharyngeal carcinoma, 18 had HPV-positive tumors and 62 were negative for HPV. The HPV status of the 22 remaining patients was unknown. HPV-positive tumors had a significantly lower ADC value compared with HPV-negative tumors, 0.81 × 10^−3^ mm^2^/s compared with 0.97 × 10^−3^ mm^2^/s (*p* < 0.01), respectively. No local recurrences were detected in the HPV-positive group and 18 recurrences occurred in the HPV-negative group. Removing the 18 HPV-positive patients from the total sample of 217 patients and retesting the predictive value of ADC and volume did not change the final conclusions.

## Importance of findings

The findings of our study imply that measuring pretreatment ADC does not help clinicians to predict a future local recurrence when T-stage is already known. This is unfortunate as a relatively easily obtainable quantitative tumor characteristic such as pretreatment ADC would be very helpful in recurrence prediction. The change in ADC (∆ADC) between pretreatment ADC and ADC values obtained during treatment might have more prognostic value [5, 7, 9].

## Discussion

In contrast to our study, an association between pretreatment ADC and local recurrence has previously been described [2–4]. Most of these studies included a substantially lower amount of patients in comparison with our study. Only the study reported by Lambrecht et al [4] had a comparable sample size. This study included 161 patients and performed multivariable analysis including, amongst others, tumor ADC and tumor volume. Additionally, similar to our methodology, they created two models, one with ADC included and one without ADC and compared the performance of the models. They report an AUC, used to determine the discriminatory capacity of the first model, of 0.62 (CI95% 0.56–0.70), while for the model without ADC, the AUC is 0.60 (CI95% 0.55–0.67). These results are very similar to our findings and it supports our conclusion that ADC has no added value as a prognostic factor for local recurrence to, more easily obtainable, clinical parameters. A full discussion of the results can be found in the supplemental material.

## Conclusion

In this study, we found pretreatment ADC to have no added value to clinical T-stage as a prognostic factor for local recurrences of HNSCC within 3 years after (chemo)radiotherapy.

## Electronic supplementary material

ESM 1 (DOCX 2744 kb)

## Abbreviations

ADC: Apparent diffusion coefficient
AUC: Area under the curve
CI95%: 95% confidence interval
(C)RT: (Chemo)radiotherapy
DW-MRI: Diffusion-weighted MRI
HNSCC: Head and neck squamous cell carcinoma
HPV: Human papilloma virus
ROC: Receiver operating characteristic
