# Supplementary material for: Pretreatment ADC is not a prognostic factor for local recurrences in head and neck squamous cell carcinoma when clinical T-stage is known
Source: Eur Radiol. 2019 Sep 16;30(2):1228–31. doi: 10.1007/s00330-019-06426-y (PMC6957548; doi:10.1007/s00330-019-06426-y)
Supplement: Supplementary file 1 — (DOCX 2744 kb) [file 330_2019_6426_MOESM1_ESM.docx]

**Supplemental Material**


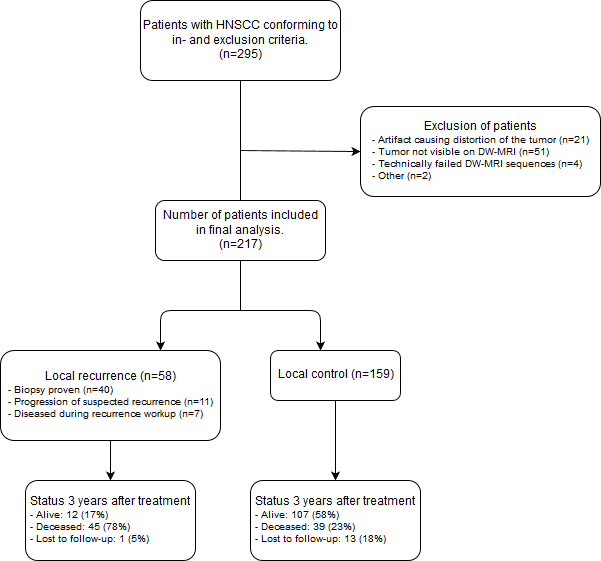


**Figure S1 Flowchart**


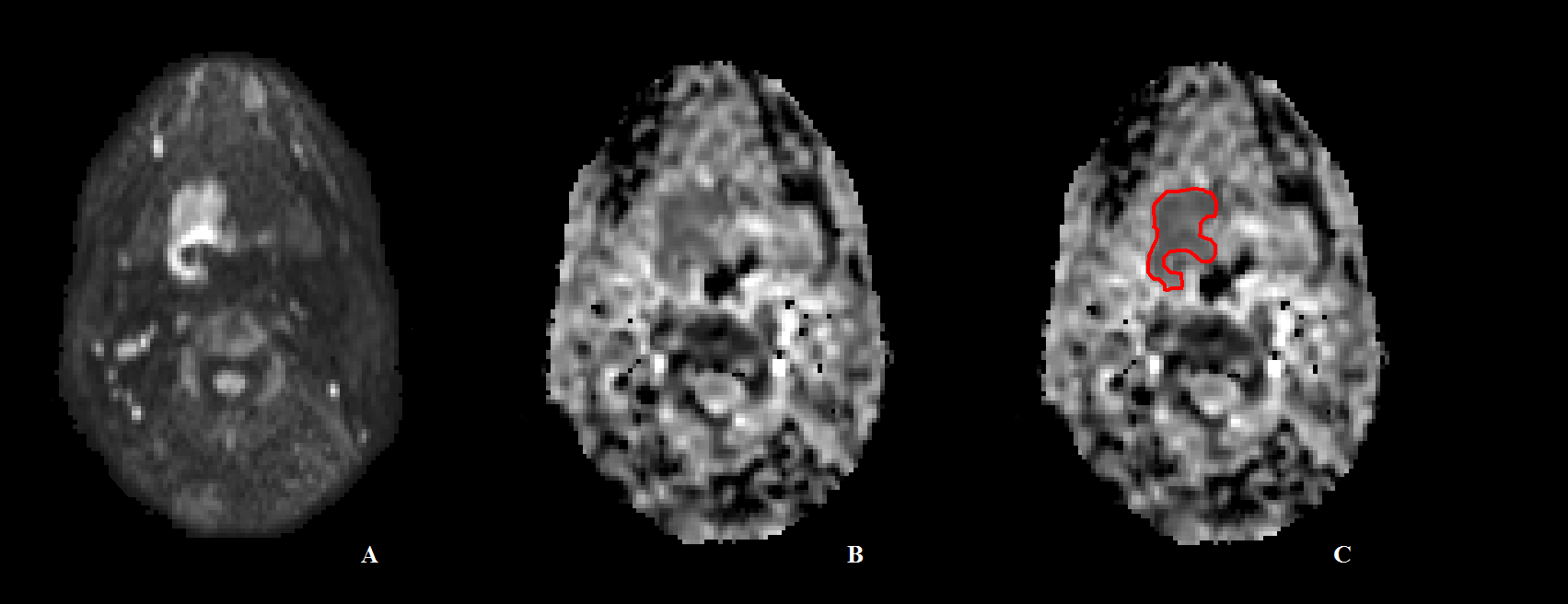


**Figure S2. Images of a patient with a T4aN2c oropharyngeal carcinoma. DW-MRI acquired in 2011. A: DW-MRI b800 s/mm^2^ image, B corresponding ADC map, C: corresponding ADC map with the contour used to determine the median ADC value of the tumor.**

**
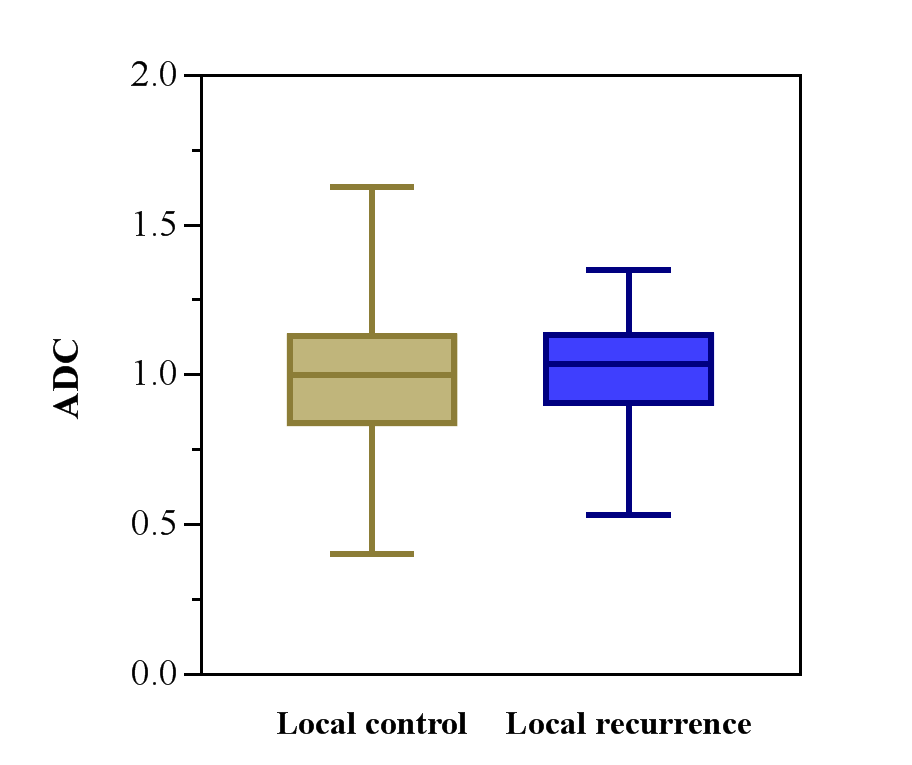
**

**Figure S3. ADC values in** **10^-3^ mm^2^/s of patients with local control and local recurrence. The box depicts the percentile range of p25-p75 and whiskers depict the range of ADC values.**

**
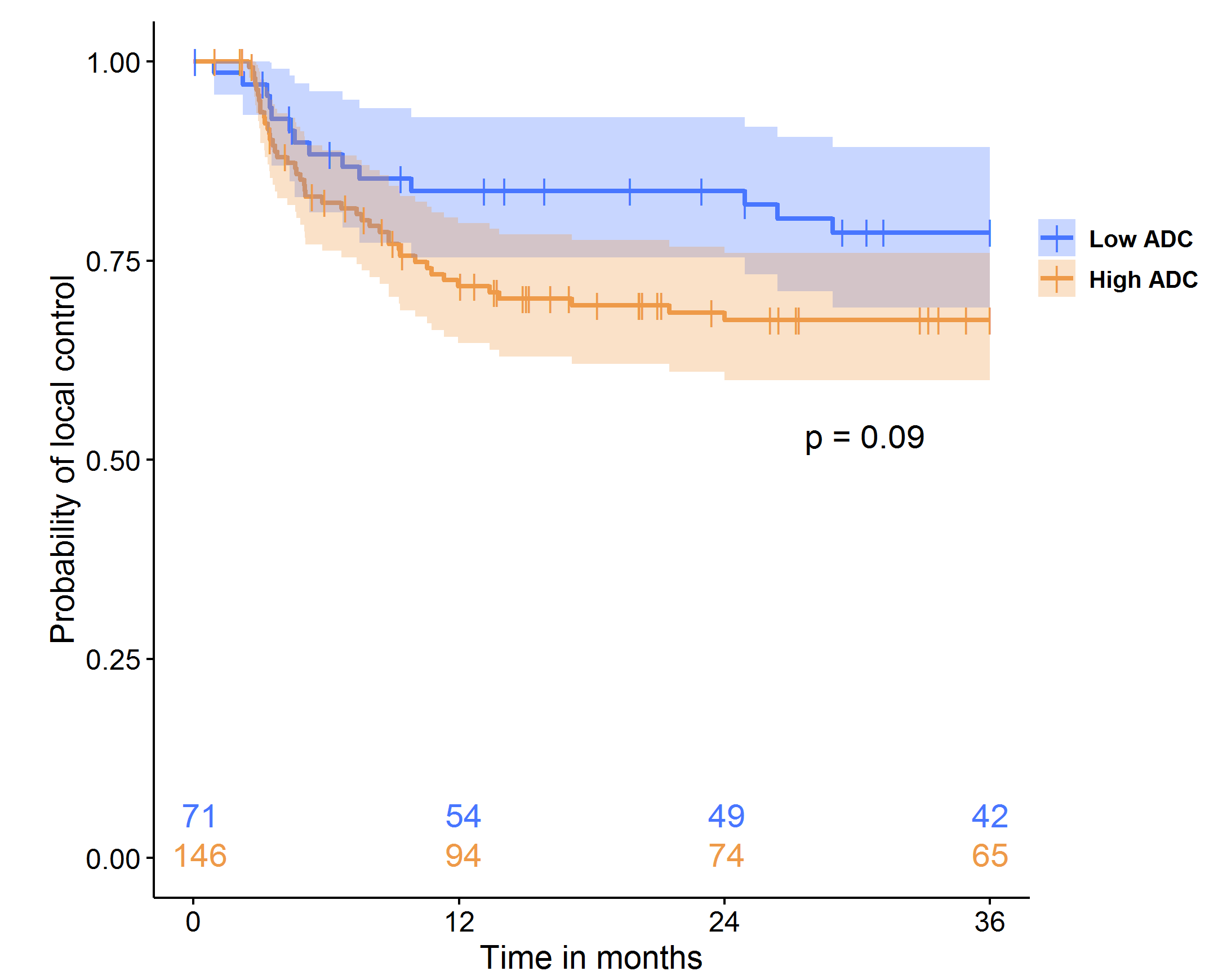
**

**Figure S4. Survival curve with CI95% showing local disease free survival of groups with ADC values higher or lower than 0.90 ∙ 10^-3^ mm^2^/s. The number at risk for each group are displayed in the lower portion of the graph.**

**
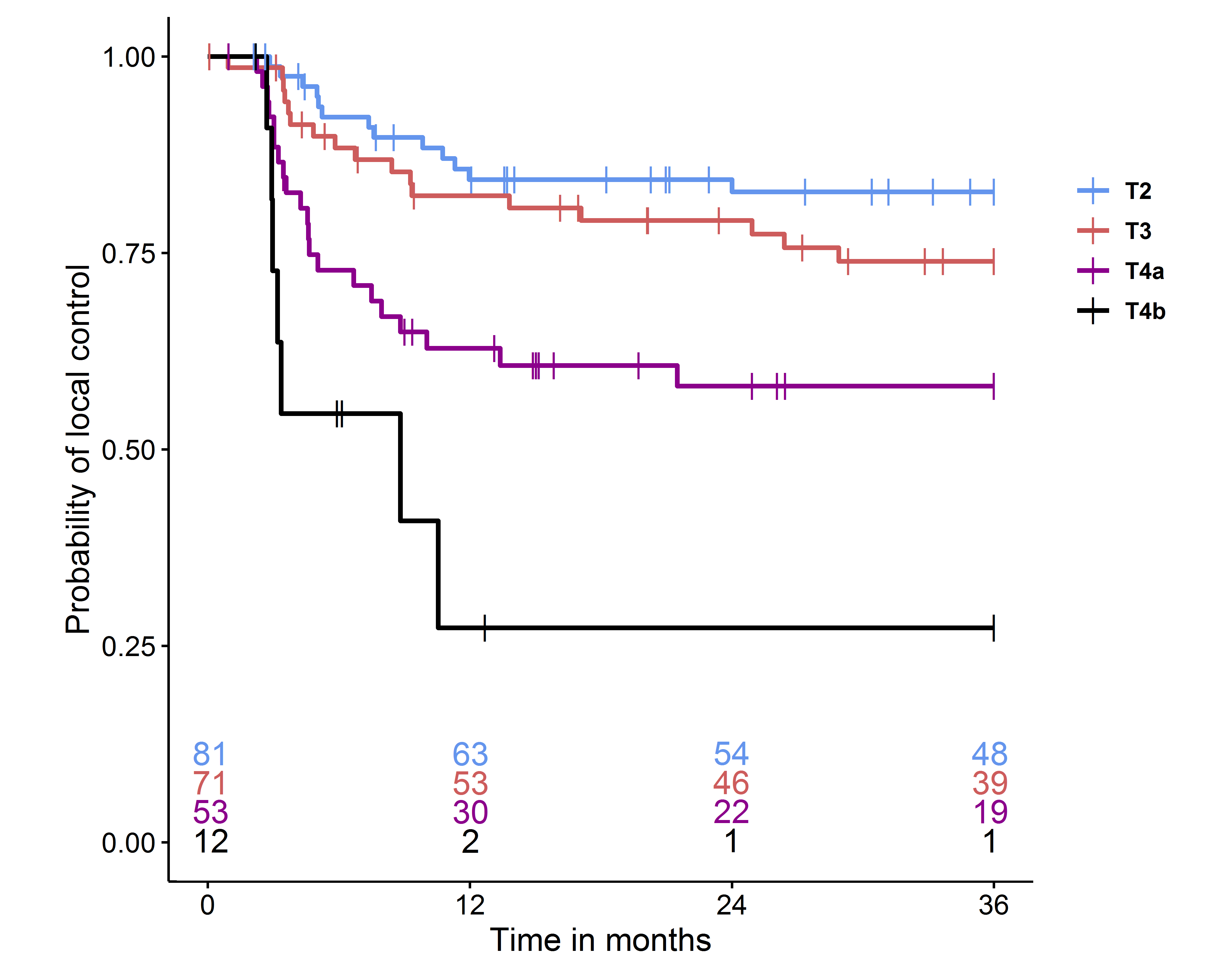
**

**Figure S5. Survival curve showing local disease free survival by T-stage. The number at risk for each group are displayed in the lower portion of the graph.**

**
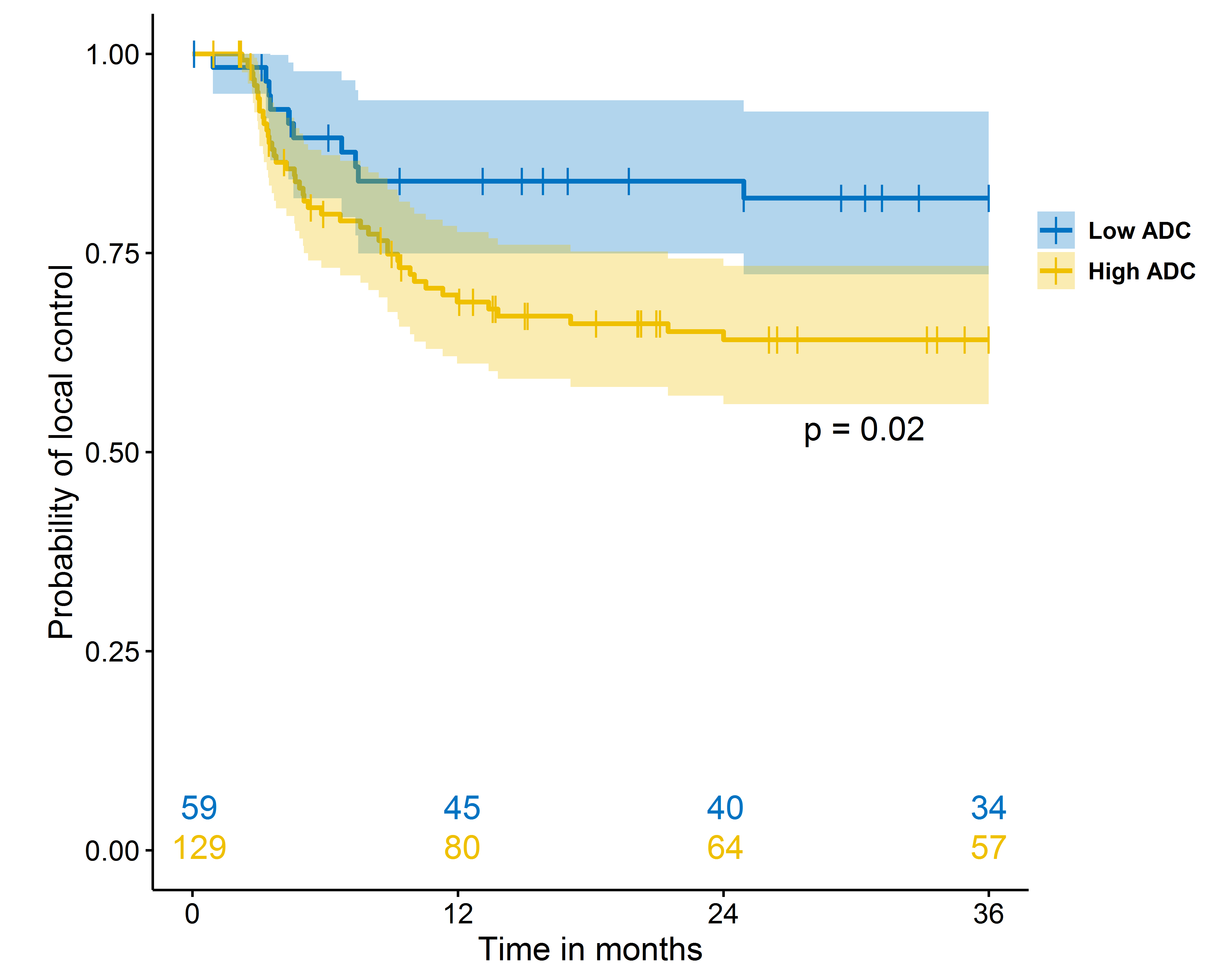
**

**Figure S6. Survival curve with CI95% showing local disease free survival using ADC values obtained in the sensitivity analysis. The ADC cutoff value separating both groups is 0.85 ∙ 10^-3^ mm^2^/s.**

|  |  |  | **Resolution (mm)** | | |
| --- | --- | --- | --- | --- | --- |
| **Sequence** | **Echo Time (ms)** | **Repetition Time (ms)** | **x** | **y** | **z** |
|  |  |  |  |  |  |
| T1 | 10 - 120 | 600 - 800 | 1 | 1 | 2 |
| T2 | 100 - 220 | 2300 - 4200 | 0.5 - 1 | 1 | 2 - 5 |
| DW-MRI | 68 - 70 | 3700 - 5900 | 1.4 - 2.2 | 1.4 - 2.5 | 3 - 4 |

**Table S1 MRI protocol detail**

| **Variable** | **Beta coefficient** | **Standard error** | **p-value** | **Hazard ratio** | **Hazard ratio CI95%** |
| --- | --- | --- | --- | --- | --- |
| T2 (reference) | .. | .. | .. | .. | .. |
| T3 | 0.44 | 0.37 | 0.24 | 1.55 | 0.75 – 3.18 |
| T4a | 1.20 | 0.35 | <0.01 | 3.31 | 1.67 – 6.57 |
| T4b | 2.05 | 0.47 | <0.01 | 7.78 | 3.08 – 19.7 |

**Table S2 Cox regression. Effect of T-stage local recurrence.**

T stage according to American Joint Committee on Cancer 7^th^ edition.

| **Variable** | **Beta coefficient** | **Standard error** | **p-value** | **Hazard ratio** | **Hazard ratio CI95%** |
| --- | --- | --- | --- | --- | --- |
| ADC | 0.26 | 0.63 | 0.69 | 1.29 | 0.37 – 4.47 |
| T2 (reference) | .. | .. | .. | .. | .. |
| T3 | 0.43 | 0.37 | 0.24 | 1.54 | 0.75 – 3.18 |
| T4a | 1.13 | 0.35 | <0.01 | 3.11 | 1.56 – 6.22 |
| T4b | 2.06 | 0.47 | <0.01 | 7.88 | 3.11 – 19.9 |

**Table S3 Cox regression. Effect of T-stage and ADC on local recurrence.**

ADC in 10^-3^ s/mm^2^. T stage according to American Joint Committee on Cancer 7^th^ edition.

| **Variable** | **Beta coefficient** | **Standard error** | **p-value** | **Hazard ratio** | **Hazard ratio CI95%** |
| --- | --- | --- | --- | --- | --- |
| ADC | 0.63 | 0.70 | 0.37 | 1.88 | 0.47 – 7.50 |
| T2 (reference) | .. | .. | .. | .. | .. |
| T3 | 0.51 | 0.41 | 0.21 | 1.67 | 0.75 – 3.73 |
| T4a | 1.14 | 0.39 | <0.01 | 3.12 | 1.47 – 6.63 |
| T4b | 2.05 | 0.50 | <0.01 | 7.80 | 2.94 – 20.7 |

**Table S4 Cox regression using reduced margins (n=188). Effect of T-stage and ADC on local recurrence.**

ADC in 10^-3^ s/mm^2^. T stage according to American Joint Committee on Cancer 7^th^ edition.

**Methods and materials**

This retrospective study was approved by the institutional review board and the need for informed consent was waived. Reporting was done in accordance to the STROBE statement [1].

*Study population*

This retrospective cohort study included consecutive patients from a single tertiary care hospital with biopsy proven primary HNSCC of the oropharynx, hypopharynx, larynx or oral cavity, who were treated with (chemo)radiotherapy with curative intent from April 2009 to December 2015. Patients were included if an MRI with diffusion-weighted sequences acquired within 2,5 months prior to the start of treatment was available.

After the diagnosis of HNSCC, all patients were discussed in a tumor board meeting to determine the stage of the tumor and the suggested treatment. The clinical T-stage was extracted from the report of this meeting for use in our study. Staging of patients was done according to the seventh edition of the American Joint Commission on Cancer [2].

Patients with T1 tumors were excluded due to reduced visibility of these tumors on MRI. Clinical charts were retrospectively reviewed for the baseline characteristics of the study population (table I).

*Treatment protocol*

Treatment consisted of 5-7 weeks of radiotherapy with an effective dose of 70 Gy to the primary tumor. Patients with an indication for concomitant chemotherapy were administered 100 mg cisplatin/m^2^ at day 1, 22 and 43. If chemotherapy with cisplatin was not possible due to severe comorbidities, patients received cetuximab instead of cisplatin. After completion of the treatment, the follow-up consisted of visits to the multidisciplinary outpatient clinic according to national guidelines; every 2 months in the first year, every 3 months in the second year after treatment, every 4 months in the third year and every 6 months in the fourth and fifth year after treatment. These consultations contained physical examination and fiberoptic endoscopy. Additional imaging was performed on indication, for example when there were complaints or clinical findings suspect for local or regional recurrence.

*Local recurrence*

Local recurrence was defined as a biopsy proven squamous cell carcinoma of the primary tumor region within three years after completion of the (chemo)radiotherapy treatment. Additionally patients clinically suspected of local recurrence within three years after treatment who did not have a (conclusive) biopsy were considered to have a local recurrence if the suspected recurrence progressed between follow up visits or if the patient died while the diagnostic workup of the suspected recurrence was still ongoing. The time to recurrence was defined as the time between the last day of therapy and the first day a suspicion of recurrence as recorded in the subject's chart.

Subjects who did not meet above criteria and who completed three years of follow up were considered to have had no local recurrence. Patients who were lost to follow up without a local recurrence as previously defined were considered to have local control for the period between end of treatment and their last follow up visit.

*MRI and diffusion-weighted MRI*

All patients underwent MRI with diffusion-weighted imaging on a 1.5 Tesla Philips Intera or 3.0 Tesla Philips Ingenia MRI scanner prior to the start of treatment. The obtained images were used for target and organs-at-risk delineation for radiotherapy treatment planning. The MRIs were acquired routinely over a period in which new protocols were developed (Table S1, in supplemental material). Available b-values differed across scans with all containing at least a high (b800 or b1000 s/mm^2^) and a low (b0 s/mm^2^) b-value. ADC maps were calculated from the DW images using all available b-values and a mono-exponential model.

*Tumor delineation*

The tumor delineation procedure was semi-automatic. The endoscopy report and available pretreatment imaging was used to determine the location of the tumor. First, a seed point was placed in the tumor on the axial DW-MRI with the highest available b value (b800 or b1000 s/mm^2^). Using the seed point and a threshold of 50% of the maximum signal intensity the tumor was segmented. Secondly, this segmentation was transferred to the corresponding ADC map. The contrast of the ADC map was set to window: 1500 x 10^-6^ mm^2^/s and level: 1000 x 10^-6^ mm^2^/s. Areas with relatively high ADC values at the outer-contour of the segmentation were considered not to be part of the tumor and were manually removed from the volume of interest by a single observer blinded to the local recurrence status of subjects. High or very low ADC values inside the tumor were not excluded to maintain tumor heterogeneity. This final segmentation was used to determine tumor volume and ADC values. The ADC value of an individual tumor was defined as the median ADC value of all voxels included in the segmentation.

*ADC and HPV*

A possible relationship exists between human papilloma virus (HPV), ADC and local recurrence in oropharyngeal carcinoma [3–6]. In order to test if this relationship had any influence on the final results ADC values of the patients with HPV positive oropharyngeal tumors were compared to those with HPV negative oropharyngeal tumors.

*Sensitivity analysis*

In order to test the robustness of the results and their susceptibility to variation in delineation a sensitivity analysis was performed. In this analysis we reduced the segmentations by 1mm in x, y and z directions. Theoretically, this reduced volume should contain a higher percentage of tumor and less of the edges of the tumor and surrounding tissues reducing partial volume effects. This reduced segmentation was used to recalculate tumor ADC and to repeat the rest of the applied analyses.

*Statistical analysis*

Differences in ADC values between patients with local control and local recurrence were determined using an independent samples t-test. The most discriminating ADC cutoff value (i.e. the value with the highest combined sensitivity and specificity) was extracted from the receiver operating characteristic (ROC) curve of ADC and local recurrence. Local disease free survival was visualized by Kaplan Meier curves using the Log-rank test to determine significant differences between the high and low ADC groups. Finally, two models were created; a Cox regression model containing T-stage as a categorical predictor and a Cox regression model containing both ADC (continuous) and T-stage. The area under the ROC curve (AUC) of both models, using probabilities for local control at 3 years, was calculated to determine the added value of ADC. P-values smaller than 0.05 were considered statistically significant. Statistical analyses were performed with SPSS (IBM Corp. Released 2015. IBM SPSS Statistics for Windows, Version 23.0., IBM Corp).

**Discussion**

In this retrospective cohort study we found that pretreatment ADC has no added value to clinical T-stage as a prognostic factor for local recurrence of HNSCC after (chemo)radiotherapy. Furthermore, in our primary analysis median ADC is not correlated with local recurrence within 3 years. Clinical T-stage however, is correlated with local recurrence, with higher stage tumors showing a higher rate of local recurrence.

The absence of an association between ADC and local recurrence did not confirm the results of previous studies, which concluded that ADC was able to predict local recurrence after (C)RT [7–9]. However, it is in line with other research which reported that local recurrence could not be predicted by pretreatment ADC [10–12]. Reports with mixed conclusion on the value of pretreatment ADC measurements exist as well [13, 14].

The conflicting results reported in previous work might be due to the different tumor delineation methods. Most studies calculated ADC on a single axial slice of the MRI (7,8). ADC values change depending on the delineation method used, this might be due to the heterogeneity of HNSCC [15]. We showed the effect of different delineation methods by removing 1 mm isotropically of the original delineation. This had two effects, first, some tumors were too small to analyze after shrinkage and had to be excluded from analysis; secondly, ADC values of all tumors changed due to the new delineations. It suggests that a hard cutoff value for ADC, reported by previous studies, might not be generalizable to other study populations unless delineation protocols are reproducible and study populations are interchangeable. For this study, we used a semi-automatic method for segmentation of the primary tumor in order to increase reproducibility, but some subjective decisions concerning the delineation are unavoidable.

Another point of difference between our study and previous work is that predominantly, these earlier studies do not take known prognostic factors such as tumor stage or tumor volume into account. This step is important as it provides clinical relevance of the added prognostic value of ADC. Clinical T-stage is arguably more easily obtained than tumor ADC and is already widely used in daily practice to determine prognosis and treatment strategies for individual patients. ADC should significantly add to this existing model in order to justify its use as a prognostic factor for local recurrence.

One study that also found pretreatment ADC to be predictive of local recurrence is the study of Lambrecht et al. [14]. This study included 161 patients and performed multivariable multivariate analysis including, amongst others, tumor ADC and tumor volume. Additionally, similar to our methodology, they created two models, one with ADC included and one without ADC and compared the performance of the models. They report an AUC, used to determine the discriminatory capacity of the first model, of 0.62 (CI 0.56 – 0.70), while for the model without ADC the AUC is 0.60 (CI 0.55 – 0.67). These result are very similar to our findings and it supports our conclusion that ADC has no added value as a prognostic factor for local recurrence to, more easily obtainable, clinical parameters.

Finally, all of these studies, except for Lambrecht et al. [14], suffered from small sample sizes. The second largest sample size was obtained by King et al. [12] who investigated 50 patients. Our study reports on a large sample size of 217 subjects and a total of 58 local recurrence.

Although the biological origin of ADC remains partly unknown, it is related to cell density, stromal components of the tumor and infiltration of inflammatory cells and also correlates to HPV status [16, 4, 17, 18]. High stromal proliferation is associated with poor outcome, and also with high ADC [19, 20]. HPV positive tumors, which are known for their prognostic favorable outcome compared to their HPV negative counterparts, have significantly lower ADC compared to HPV negative tumors [4, 6, 5]. This was confirmed in our study and suggests that ADC could be able to serve as a predictor of outcome but only as a surrogate marker for HPV status. This might have biased studies which showed a predictive value for ADC but failed to correct for HPV status.

An interesting alternative to pretreatment ADC as a predictor of local recurrence might be the change in ADC (∆ADC) between pretreatment ADC and ADC values obtained during treatment. Some studies report that this ∆ADC has prognostic power, theorizing that regardless of pretreatment ADC values, different tumors respond differently to (C)RT and that this effect can only be determined after treatment has started [12, 21].

We used clinical T-stage as a variable to compare ADC to. This study confirms that this seemingly straightforward model, based on clinical observations, is able to differentiate patients at risk for local recurrence from patients with local control reasonably well.

*Limitations*

Our study had some limitations. We excluded many patients due to insufficient visibility of the tumor on DW-MRI. Furthermore, the use of different DW-MRI protocols might influence our measurement of ADC values [22]. In our retrospective study the DW-MRI protocols changed over the years. This might result in different ADC values for similar tumors, possibly obscuring the prognostic value of ADC. Nonetheless, the use of different DW-MRI protocols results in a model applicable to clinical practice. Finally, the obtained results were not validated in an external cohort, yet the similarity in results between our study and that of the only other large sample size study supports our findings [14].

**References**

1. Vandenbroucke JP, von Elm E, Altman DG, et al (2007) Strengthening the Reporting of Observational Studies in Epidemiology (STROBE): explanation and elaboration. PLoS Med 4:e297 . doi: 10.1371/journal.pmed.0040297

2. Edge SB, Compton CC (2010) The American Joint Committee on Cancer: the 7th edition of the AJCC cancer staging manual and the future of TNM. Ann Surg Oncol 17:1471–4 . doi: 10.1245/s10434-010-0985-4

3. Ravanelli M, Grammatica A, Tononcelli E, et al (2018) Correlation between Human Papillomavirus Status and Quantitative MR Imaging Parameters including Diffusion-Weighted Imaging and Texture Features in Oropharyngeal Carcinoma. AJNR Am J Neuroradiol 39:1878–1883 . doi: 10.3174/ajnr.A5792

4. Driessen JP, van Bemmel AJM, van Kempen PMW, et al (2015) Correlation of human papillomavirus status with apparent diffusion coefficient of diffusion-weighted MRI in head and neck squamous cell carcinomas. Head Neck 38:613–8 . doi: 10.1002/hed.24051

5. Kimple RJ, Smith MA, Blitzer GC, et al (2013) Enhanced radiation sensitivity in HPV-positive head and neck cancer. Cancer Res 73:4791–800 . doi: 10.1158/0008-5472.CAN-13-0587

6. Isayeva T, Li Y, Maswahu D, Brandwein-Gensler M (2012) Human papillomavirus in non-oropharyngeal head and neck cancers: a systematic literature review. Head Neck Pathol 6 Suppl 1:S104-20 . doi: 10.1007/s12105-012-0368-1

7. Hatakenaka M, Nakamura K, Yabuuchi H, et al (2011) Pretreatment apparent diffusion coefficient of the primary lesion correlates with local failure in head-and-neck cancer treated with chemoradiotherapy or radiotherapy. Int J Radiat Oncol Biol Phys 81:339–345 . doi: 10.1016/j.ijrobp.2010.05.051

8. Hatakenaka M, Shioyama Y, Nakamura K, et al (2011) Apparent diffusion coefficient calculated with relatively high b-values correlates with local failure of head and neck squamous cell carcinoma treated with radiotherapy. AJNR Am J Neuroradiol 32:1904–1910 . doi: 10.3174/ajnr.A2610

9. Ohnishi K, Shioyama Y, Hatakenaka M, et al (2011) Prediction of local failures with a combination of pretreatment tumor volume and apparent diffusion coefficient in patients treated with definitive radiotherapy for hypopharyngeal or oropharyngeal squamous cell carcinoma. J Radiat Res 52:522–530

10. Chawla S, Kim S, Dougherty L, et al (2013) Pretreatment diffusion-weighted and dynamic contrast-enhanced MRI for prediction of local treatment response in squamous cell carcinomas of the head and neck. AJR Am J Roentgenol 200:35–43 . doi: 10.2214/AJR.12.9432

11. King AD, Chow K-K, Yu K-H, et al (2013) Head and neck squamous cell carcinoma: diagnostic performance of diffusion-weighted MR imaging for the prediction of treatment response. Radiology 266:531–538 . doi: 10.1148/radiol.12120167

12. King AD, Mo FKF, Yu K-H, et al (2010) Squamous cell carcinoma of the head and neck: diffusion-weighted MR imaging for prediction and monitoring of treatment response. Eur Radiol 20:2213–2220 . doi: 10.1007/s00330-010-1769-8

13. Lombardi M, Cascone T, Guenzi E, et al (2017) Predictive value of pre-treatment apparent diffusion coefficient (ADC) in radio-chemiotherapy treated head and neck squamous cell carcinoma. Radiol Med 122:345–352 . doi: 10.1007/s11547-017-0733-y

14. Lambrecht M, Van Calster B, Vandecaveye V, et al (2014) Integrating pretreatment diffusion weighted MRI into a multivariable prognostic model for head and neck squamous cell carcinoma. Radiother Oncol 110:429–434 . doi: 10.1016/j.radonc.2014.01.004

15. Perrot XT De, Lenoir X V, Ayllo XMD, et al (2017) Apparent Diffusion Coefficient Histograms of Human Papillomavirus – Positive and Human Papillomavirus – Negative Head and Neck Squamous Cell Carcinoma : Assessment of Tumor Heterogeneity and Comparison with Histopathology. 2153–2160

16. Driessen JP, Caldas-Magalhaes J, Janssen LM, et al (2014) Diffusion-weighted MR imaging in laryngeal and hypopharyngeal carcinoma: association between apparent diffusion coefficient and histologic findings. Radiology 272:456–463 . doi: 10.1148/radiol.14131173

17. Sun Y, Tong T, Cai S, et al (2014) Apparent Diffusion Coefficient (ADC) value: a potential imaging biomarker that reflects the biological features of rectal cancer. PLoS One 9:e109371 . doi: 10.1371/journal.pone.0109371

18. Choi SY, Chang Y-W, Park HJ, et al (2012) Correlation of the apparent diffusion coefficiency values on diffusion-weighted imaging with prognostic factors for breast cancer. Br J Radiol 85:e474-9 . doi: 10.1259/bjr/79381464

19. de Kruijf EM, van Nes JGH, van de Velde CJH, et al (2011) Tumor-stroma ratio in the primary tumor is a prognostic factor in early breast cancer patients, especially in triple-negative carcinoma patients. Breast Cancer Res Treat 125:687–96 . doi: 10.1007/s10549-010-0855-6

20. Wang K, Ma W, Wang J, et al (2012) Tumor-stroma ratio is an independent predictor for survival in esophageal squamous cell carcinoma. J Thorac Oncol 7:1457–61 . doi: 10.1097/JTO.0b013e318260dfe8

21. Vandecaveye V, Dirix P, De Keyzer F, et al (2010) Predictive value of diffusion-weighted magnetic resonance imaging during chemoradiotherapy for head and neck squamous cell carcinoma. Eur Radiol 20:1703–1714 . doi: 10.1007/s00330-010-1734-6

22. Kolff-Gart AS, Pouwels PJW, Noij DP, et al (2015) Diffusion-weighted imaging of the head and neck in healthy subjects: reproducibility of ADC values in different MRI systems and repeat sessions. AJNR Am J Neuroradiol 36:384–90 . doi: 10.3174/ajnr.A4114
